# Comparison of risk factors and outcomes of gestational hypertension and pre-eclampsia

**DOI:** 10.1371/journal.pone.0175914

**Published:** 2017-04-24

**Authors:** Minxue Shen, Graeme N. Smith, Marc Rodger, Ruth Rennicks White, Mark C. Walker, Shi Wu Wen

**Affiliations:** 1 Department of Dermatology, Xiangya Hospital, Central South University, Changsha, Hunan, China; 2 Department of Epidemiology and Health Statistics, Xiangya School of Public Health, Central South University, Changsha, Hunan, China; 3 OMNI Research Group, Department of Obstetrics and Gynecology, Faculty of Medicine, University of Ottawa, Ottawa, Ontario, Canada; 4 Ottawa Hospital Research Institute, Clinical Epidemiology Program, Ottawa, Ontario, Canada; 5 Department of Obstetrics and Gynecology, Queen’s University, Kingston, Ontario, Canada; 6 School of Epidemiology, Public Health, and Preventive Medicine, Faculty of Medicine, University of Ottawa, Ottawa, Ontario, Canada; 7 Department of Medicine, Faculty of Medicine, University of Ottawa, Ottawa, Ontario, Canada; Helsingin Yliopisto, FINLAND

## Abstract

**Background:**

It remains an enigma whether gestational hypertension (GH) and pre-eclampsia (PE) are distinct entities or different spectrum of the same disease. We aimed to compare the risk factors and outcomes between GH and PE.

**Method:**

A total of 7,633 pregnant women recruited between 12 and 20 weeks of gestation in the Ottawa and Kingston Birth Cohort from 2002 to 2009 were included in the analysis. Cox proportional hazards model was used to identify and compare the risk factors for GH and PE by treating gestational age at delivery as the survival time. Logistic regression model was used to compare outcome. Subgroup analysis was performed for early- and late-onset PE.

**Results:**

GH and PE shared most risk factors including overweight and obesity, nulliparity, PE history, type 1 and 2 diabetes, and twin birth. Effect size of PE history (RR = 14.1 for GH vs. RR = 6.4 for PE) and twin birth (RR = 4.8 for GH vs. RR = 10.3 for PE) showed substantial difference. Risk factors modified gestational age at delivery in patients with GH and PE in similar pattern. Subgroup analysis showed that early- and late-onset PE shared some risk factors with different effect sizes, whereas folic acid supplementation showed protective effect for early-onset PE only. PE was strongly associated with several adverse outcomes including cesarean section, placental abruption, small for gestational age, preterm birth, and 5 min Apgar score < 7, whereas GH was associated with increased risk of preterm birth only.

**Conclusions:**

GH and PE shared common risk factors. Differences in effect sizes of risk factors and outcomes indicate that the conditions may have different pathophysiology and mechanism.

## Introduction

Hypertensive disorders of pregnancy (HDP) remain a major health issue for women and their descendants worldwide [1]. Pre-eclampsia (PE) complicates 2–5% of all pregnancies and is a leading cause of maternal and neonatal mortality and morbidity [2]. PE and eclampsia are estimated to be responsible for approximately 14% of maternal death [3]. PE is also associated with later-life cardiovascular disease among women and their offspring [4]. Gestational hypertension (GH) affects 5–10% of pregnancies, but its complications are less severe [5]. Different from pre-existing hypertension with or without superimposed PE, both GH and PE are characterized by de novo hypertension developing in pregnancy [1]. In addition, PE is defined by new-onset proteinuria or other end-organ dysfunctions.

It still remains an enigma whether GH and PE are distinct entities with shared manifestations, or different spectrum of the same disease. In many PE patients, the occurrence of hypertension precedes proteinuria or other features of systematic disorders; on the other hand, GH does not necessarily develops into PE [6]. Previous epidemiological studies examined the risk factors of GH and PE, providing useful information with respect to the understanding of potential etiologic mechanisms as well as prediction and management of HDP [7–9]. However, most studies were focused on PE because of the clinical importance. Few studies compared the risk factors of GH and PE within the same population [10–14], and their findings were inconsistent. The inconsistency of findings may be attributable to different diagnostic criteria, heterogeneity of population, and sample size. Many of these studies did not exclude women with chronic hypertension and superimposed PE [10–12].

In recognition of the syndromic nature of PE, both the American College of Obstetricians and Gynecologists (ACOG) as well as the Society of Obstetricians and Gynecologists of Canada (SOGC) have modified the diagnostic criteria for PE in recent years [1, 15]. They have eliminated the dependence of PE diagnosis on proteinuria. The changes in diagnostic criteria and categorization demonstrate limited understanding on the etiologic differences between GH and PE. A novel approach to investigate the risk factors for HDP was proposed by Wright *et al* recently [16]. It is a survival-time model that treats gestational age (GA) at the time of delivery as a continuous variable, and assumes that all women would develop PE if the pregnancy was to continue indefinitely [16]. The occurrence of disease depends on competition between delivery before or after the development of PE [16]. Under this hypothesis, risk factors can be interpreted as the effects to modify the average GA in women with HDP. In low-risk women, the distribution of GA at delivery is shifted to the right with the implication that in most pregnancies, delivery will occur before the development of HDP. Based on this method, a screening tool for the early prediction of PE with acceptable performance has been proposed [17].

The aforementioned studies compared the risk factors but not outcomes between GH and PE in European, Asian and South American populations using the proteinuria-dependent diagnostic criteria of PE. Statistical approach used in these studies was conventional logistic regression model. We found it necessary to replicate the comparison of GH and PE with respect to risk factors as well as outcomes within the same population, using the proteinuria-independent diagnostic criteria of PE. Under the primary hypothesis that GH and PE are the same disorder of progress, similarities in risk factors and their modification effects on GA at delivery may indicate common etiology, while differences in magnitude of effects of certain risk factors and outcomes may reflect different prognosis that corresponds to the deteriorating multi-organ functions in a dynamic process of the disorder.

In this current study, we investigated and compared the risk factors and the maternal and perinatal outcomes between GH and PE among women without chronic hypertension in the Ottawa and Kingston (OaK) Birth Cohort [18], based on Cox proportional hazards model that is independent of the distribution of GA at delivery.

## Methods

### Data source

The OaK Birth Cohort recruited pregnant women at The Ottawa Hospital and Kingston General Hospital from September 2002 to April 2009. Participants were recruited between 12 and 20 weeks of gestation and later delivered in the Ottawa-Carleton and Kingston regions. Pregnant women were referred to the research team by clinic staff and asked to participate at their first antenatal visit and were recruited into the study after the information about the purpose of the study was provided.

At the time of recruitment, participants’ demographic and life-style information (age, race, education, household income, folic acid supplementation, smoking, pre-gravid height and weight) as well as medical history (parity, chronic hypertension, diabetes, PE history, medication) were collected by medical chart and structured interview. Height and weight were measured by the research nurse at recruitment. Delivery and other clinical data were collected within 24–72 hours post-delivery, and a chart view was conducted to collect additional clinical data. If data was missing, the nurses called the participating women within one week after childbirth to collect the missing data.

GH was defined as a blood pressure ≥ 140 mmHg systolic or ≥ 90 mmHg diastolic on two occasions six hours apart, without the presence of proteinuria or other features of systematic disorders, after 20 weeks of gestation in a woman with a normal pre-gravid blood pressure. PE was defined as new onset hypertension with new-onset proteinuria ≥ 2+ on dipstick or ≥ 300 mg in 24 hour urine collection; in the absence of proteinuria, with the features of clinical signs and symptoms including HELLP (hemolysis, elevated liver enzymes, and low platelet count), thrombocytopenia, impaired liver function, renal insufficiency, pulmonary edema, or cerebral or visual symptoms [19]. To ensure the quality of diagnosis, adjudication by study investigators was conducted to verify the diagnosis for all cases of PE. Early-onset PE was defined as requiring delivery before 34 weeks of gestation.

Maternal and perinatal outcomes included type of delivery (cesarean or vaginal), placental abruption, small for gestational age (SGA), preterm birth, and 5 min Apgar score. SGA was defined as gestational age and gender-specific birth weight < 10^th^ percentile based on Canadian reference [20]. Preterm birth was defined as GA at delivery < 37 weeks.

### Statistical analysis

Maternal age at recruitment was categorized into two groups: < 35 and ≥ 35 years. Race was dichotomized into Caucasian and other races. Educational level was dichotomized into grade/high school, and college/university. Household income (in Canadian dollar) was grouped into ≤ 49999, 50000–79999, ≥ 80000, and declined. Body mass index (BMI) at recruitment was calculated as weight (kg) / [height (m)]^2^, and was grouped into underweight (< 18.5), normal (18.5–24.9), overweight (25–29.9) and obese (≥ 30). 5 min Apgar score was dichotomized using the cut-off value 7.

The incidence rates of GH and PE were described according to the characteristics of participants, including maternal age, race, education, household income, smoking, folic acid supplementation, BMI group, parity and PE history, conception (spontaneous vs. assisted reproductive technology, ART), diabetes (type1, type 2, and gestational diabetes), infant sex, and number of babies (singleton vs. twin birth).

Cox proportional hazards model was used to estimate effect size of risk factors. Cox regression is a semi-parametric approach, and it is independent of the distribution of survival time (GA at delivery in our study). Deliveries from causes other than GH and PE were treated as censored data. Crude relative risk (RR) and 95% confidence intervals (CI) of the aforementioned risk factors for GH and PE were estimated. Adjusted RRs and 95% CIs were obtained, adjusting for all other variables listed above. Both crude RRs and adjusted RRs were estimated by comparing to the unaffected women (without HDP). Survival curves were presented to demonstrate how risk factors modified mean GA at delivery in women with GH or PE. Subgroup analysis was performed for the early- and late-onset pre-eclampsia.

The incidences of maternal and perinatal outcomes were compared between exposure group (GH or PE) and non-exposure group (unaffected). Crude ORs, adjusted ORs and their 95% CIs were estimated from unconditional logistic regression model. Supplemental analysis was performed using pre-gravid BMI (self-reported) instead of BMI measured at recruitment. The level of significance was set at 0.05. All statistical analysis were performed using SAS 9.4 (SAS Institute Inc., Cary, North Carolina, USA).

## Results

A total of 8085 women were recruited to the OaK Birth Cohort from September 2002 to April 2009. Among them, 452 women were excluded from the analysis: 126 were patients with chronic hypertension, 130 were aborted (miscarriage / termination, < 20 weeks), and 196 had missing delivery data, leaving 7633 women for final analysis.

Mean age of the women at recruitment was 30.3 ± 5.0 years, mean BMI at recruitment was 25.8 ± 5.5 kg/m^2^, and median gestational age at delivery was 39.4 (interquartile range 2.1) weeks. 280 (3.7%) women were diagnosed as GH, and 222 (2.9%) were diagnosed as PE. 33 had early-onset PE (delivery before 34 weeks of gestation) and 189 had late-onset PE. The average gestational age in unaffected, GH, and PE group were 39.2, 38.8, and 36.9, respectively, with significant between-group differences. Table 1 shows the distribution of GH and PE cases in terms of characteristics of the participants. The incidence rates of GH and PE both varied among BMI groups, parity, and history of PE.

**Table 1:** Incidence of gestational hypertension and pre-eclampsia by characteristics of participants in the OaK birth cohort.

	Overall	GH		PE	
		N (%)	*P*	N (%)	*P*
Maternal age (years)					
< 35	6033	222 (3.7)	0.92	169 (2.8)	0.28
≥ 35	1600	58 (3.6)		53 (3.3)	
Race					
Caucasian	5938	212 (3.6)	0.39	164 (2.8)	0.15
Other races	1695	68 (4.0)		58 (3.4)	
Education					
Grade / high school	1111	28 (2.5)	0.03	34 (3.1)	0.73
College / university	6515	252 (3.9)		187 (2.9)	
Household income ($CAN)					
≤ 49999	1582	50 (3.2)	0.08	56 (3.5)	0.10
50000–79999	2078	76 (3.7)		62 (3.0)	
≥ 80000	3486	144 (4.1)		97 (2.8)	
Declined	487	10 (2.1)		7 (1.4)	
Smoking					
No	6775	253 (3.7)	0.39	199 (2.9)	0.67
Yes	858	27 (3.1)		23 (2.7)	
Folic acid supplementation					
No	398	8 (2.0)	0.07	15 (3.8)	0.30
Yes	7159	269 (3.8)		205 (2.9)	
BMI (kg/m^2^)					
< 18.5	143	1 (0.7)	<0.01	3 (2.1)	<0.01
18.5–24.9	3920	93 (2.4)		67 (1.7)	
25–29.9	2031	93 (4.6)		70 (3.4)	
≥ 30	1337	86 (6.4)		75 (5.6)	
Parity					
Nulliparous	3826	180 (4.7)	<0.01	149 (3.9)	<0.01
Parous without previous PE	3597	61 (1.7)		54 (1.5)	
Parous with previous PE	210	39 (18.6)		19 (9.0)	
Conception					
Spontaneous	7278	266 (3.7)	0.67	200 (2.7)	<0.01
Through ART	342	14 (4.1)		21 (6.1)	
Diabetes					
No	7503	273 (3.6)	0.48	207 (2.8)	<0.01
Type 1 diabetes	57	2 (3.5)		8 (14.0)	
Type 2 diabetes	52	4 (7.7)		6 (11.5)	
Gestational diabetes	21	1 (4.8)		1 (4.8)	
Infant gender					
Male	3681	144 (3.9)	0.28	104 (2.8)	0.66
Female	3942	136 (3.5)		118 (3.0)	
Number of babies					
Singleton	7513	277 (3.7)	0.57	207 (2.8)	<0.01
Twin birth	112	3 (2.7)		15 (13.4)	

GH: gestational hypertension. PE: pre-eclampsia. BMI: body mass index. ART: assisted reproductive technology.

Table 2 shows the crude RRs and adjusted RRs (ARRs) of the risk factors for GH and PE, respectively. According to the ARRs estimated by the Cox proportional hazards model, GH and PE shared all risk factors, including BMI ≥ 25, nulliparity, PE history in parous women, type 1 diabetes, type 2 diabetes, and twin birth. Effect sizes of PE history (14.1 for GH vs. 6.4 for PE) and twin birth (4.8 for GH vs. 10.3 for PE) were substantially different. Maternal age, race, education, household income, smoking, folic acid supplementation, conception through ART, and infant sex were not significant risk factors for either GH or PE (those who declined reporting household income had lower incidence of PE with marginal significance).

**Table 2:** Comparison of risk factors for gestational hypertension and pre-eclampsia.

	GH			PE		
	Crude RR [95% CI]	Adjusted RR [95% CI] ^a^	*P* ^b^	Crude RR [95% CI]	Adjusted RR [95% CI] ^a^	*P* ^b^
Age (years) ≥ 35	1.05 [0.78, 1.40]	0.95 [0.69, 1.29]	0.72	1.16 [0.5, 1.59]	1.21 [0.86, 1.68]	0.28
Caucasian	0.88 [0.6, 1.16]	0.75 [0.56, 1.00]	0.05	0.76 [0.56, 1.01]	0.76 [0.55, 1.05]	0.09
College / university education	1.54 [1.04, 2.29]	1.40 [0.87, 2.12]	0.18	0.96 [0.66, 1.39]	0.93 [0.61, 1.43]	0.74
Income ($CAN)						
≤ 49,999	1.00	1.00		1.00	1.00	
50,000–79,999	1.16 [0.81, 1.66]	1.02 [0.70, 1.50]	0.90	0.83 [0.58, 1.19]	0.91 [0.61, 1.36]	0.65
≥ 80,000	1.31 [0.95, 1.82]	1.29 [0.90, 1.86]	0.17	0.78 [0.56, 1.09]	0.90 [0.61, 1.33]	0.58
Declined	0.67 [0.34, 1.32]	0.72 [0.36, 1.42]	0.34	0.41 [0.19, 0.90]	0.42 [0.18, 0.97]	0.04
Smokers	0.92 [0.62, 1.37]	0.99 [0.64, 1.54]	0.97	0.96 [0.62, 1.47]	0.80 [0.50, 1.30]	0.37
Folic acid supplementation users	1.81 [0.90, 3.67]	1.42 [0.70, 2.90]	0.34	0.82 [0.48, 1.41]	0.66 [0.38, 1.13]	0.13
BMI (kg/m^2^)						
< 18.5	0.31 [0.04, 2.19]	0.34 [0.05, 2.45]	0.29	1.25 [0.39, 3.98]	1.26 [0.40, 4.04]	0.69
18.5–24.9	1.00	1.00		1.00	1.00	
25–29.9	1.87 [1.40, 2.49]	1.80 [1.35, 2.41]	<0.01	2.02 [1.44, 2.83]	1.93 [1.37, 2.70]	<0.01
≥ 30	2.91 [2.17, 3.91]	2.81 [2.07, 3.81]	<0.01	3.49 [2.50, 4.87]	3.38 [2.40, 4.76]	<0.01
Parity						
Nulliparous	2.41 [1.80, 3.24]	2.59 [1.90, 3.52]	<0.01	2.51 [1.83, 3.43]	2.78 [2.00, 3.86]	<0.01
Parous without previous PE	1.00	1.00		1.00	1.00	
Parous with previous PE	15.02 [10.00, 22.57]	14.09 [9.28, 21.40]	<0.01	8.59 [5.09, 14.52]	6.35 [3.69, 10.94]	<0.01
Conceived through ART	1.45 [0.85, 2.49]	1.04 [0.58, 1.86]	0.84	2.71 [1.73, 4.26]	1.27 [0.74, 2.18]	0.39
Diabetes						
Type 1 diabetes	4.83 [1.19, 19.56]	4.60 [1.12, 18.92]	0.03	11.26 [5.52, 23.00]	6.47 [3.02, 13.87]	<0.01
Type 2 diabetes	6.63 [2.46, 17.90]	3.24 [1.17, 8.97]	0.02	7.53 [3.33, 17.03]	3.76 [1.62, 8.71]	<0.01
Gestational diabetes	2.93 [0.41, 20.88]	1.41 [0.20, 10.22]	0.73	2.72 [0.38, 19.40]	0.85 [0.12, 6.25]	0.88
Male infant	0.90 [0.71, 1.14]	0.88 [0.69, 1.12]	0.30	1.05 [0.81, 1.37]	1.05 [0.80, 1.37]	0.74
Twin birth	6.21 [1.97, 19.59]	4.82 [1.47, 15.83]	0.01	15.79 [9.21, 27.06]	10.25 [5.48, 19.15]	<0.01

GH: gestational hypertension. PE: pre-eclampsia. RR: relative risk. BMI: body mass index. ART: assisted reproductive technology.

^a^ Compared with unaffected women; adjusted for all other variables listed in the table.

^b^ *P* value for adjusted RR, estimated from Cox proportional hazards model.

**Fig 1:**
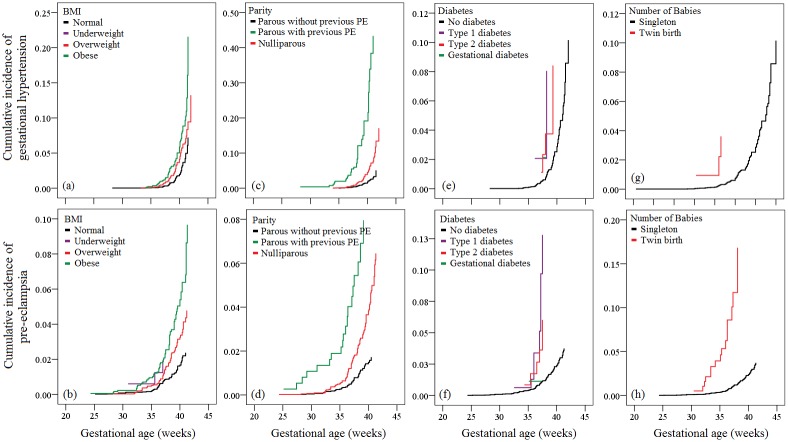
Survival curves for cumulative incidences of gestational hypertension and pre-eclampsia, stratified by common risk factors. Survival curves stratified by BMI (a, b), parity and pre-eclampsia history (c, d), diabetes (e, f) and number of babies (g, h), adjusted by age, race, education, household income, smoking, folic acid supplementation, BMI, parity, pre-eclampsia history, conception through assisted reproductive technologies, diabetes, infant sex, and number of babies. Risk factors modified the average GA in women with GH and PE in similar patterns. The survival curves were left-shifted in high-risk women (overweight or obese, nulliparous, or with PE history) compared with those in low-risk women (normal-weight, parous, or without PE history). The curves for PE were also left-shifted compared with the curves for GH.

Fig 1 shows how risk factors modify gestational age at delivery in women with GH or PE. Generally, women at high risk of GH or PE (overweight, obesity, nulliparity, PE history, type 1 diabetes, type 2 diabetes, and twin birth) had left-shifted curves (i.e. shorter mean GA at delivery) compared with women at low risk of GH or PE (normal-weight, without previous PE, without diabetes, singleton birth). Within the same category of a risk factor, the curve of PE was shifted to the left compared with the curve of GH.

Subgroup analysis for early- and late-onset PE is shown in Table 3. Early- and late-onset PE shared risk factors including BMI, parity, PE history, and twin birth. Folic acid supplementation showed a protective effect for early-onset PE but not for late-onset PE. Effect of diabetes was not estimable for early-onset PE owing to limited number of cases. The incidence rates of neither early- nor late-onset PE were associated with infant sex, and ART. Specifically, PE history had substantially stronger effect on early-onset PE than late-onset PE.

**Table 3:** Comparison of risk factors for early- and late-onset pre-eclampsia.

	Early-onset PE		Late-onset PE	
	Adjusted RR [95% CI] ^a^	*P* ^b^	Adjusted RR [95% CI] ^a^	*P* ^b^
Age (years) ≥ 35	1.16 [0.50, 2.71]	0.73	1.27 [0.89, 1.83]	0.19
Caucasian	0.67 [0.30, 1.48]	0.32	0.78 [0.55, 1.10]	0.16
College / university education	1.09 [0.53, 2.25]	0.31	0.84 [0.54, 1.33]	0.46
Income ($CAN)				
≤ 49,999	1.00		1.00	
50,000–79,999	0.80 [0.28, 2.35]	0.69	0.90 [0.59, 1.39]	0.64
≥ 80,000	1.03 [0.37, 2.89]	0.95	0.84 [0.55, 1.28]	0.42
Declined	0.50 [0.06, 4.11]	0.52	0.40 [0.16, 0.99]	0.05
Smokers	1.65 [0.58, 4.67]	0.35	0.67 [0.39, 1.15]	0.15
Folic acid supplementation users	0.33 [0.11, 0.99]	0.04	0.80 [0.43, 1.48]	0.47
BMI (kg/m^2^)				
< 18.5	4.45 [0.54, 36.57]	0.317	0.92 [0.22, 3.78]	0.91
18.5–24.9	1.00		1.00	
25–29.9	2.15 [0.85, 5.44]	0.11	1.89 [1.31, 2.72]	<0.01
≥ 30	4.03 [1.60, 10.16]	<0.01	3.22 [2.22, 4.66]	<0.01
Parity				
Nulliparous	2.23 [0.93, 5.36]	0.07	2.89 [2.03, 4.13]	<0.01
Parous without previous PE	1.00		1.00	
Parous with previous PE	16.98 [6.01, 47.96]	<0.01	4.88 [2.53, 9.40]	<0.01
Conceived through ART	2.44 [0.781, 7.37]	0.11	1.05 [0.56, 1.98]	0.88
Diabetes				
Type 1 diabetes	5.35 [0.69, 41.65]	0.11	9.29 [4.17, 20.72]	<0.01
Type 2 diabetes	N/A		5.41 [2.32, 12.63]	<0.01
Gestational diabetes	N/A		1.26 [0.17, 9.31]	0.82
Male infant	1.20 [0.59, 2.46]	0.61	1.01 [0.75, 1.35]	0.96
Twin birth	12.05 [4.10, 35.41]	<0.01	10.22 [4.66, 22.38]	<0.01

PE: pre-eclampsia. RR: relative risk. BMI: body mass index. ART: assisted reproductive technology. N/A: not estimable.

^a^ Compared with unaffected women; adjusted for all other variables listed in the table.

^b^ *P* value for adjusted RR, estimated from Cox proportional hazards model.

Table 4 shows the maternal and perinatal outcomes in association with GH and PE. PE significantly increased the risk of adverse outcomes including cesarean section (AOR = 2.2), placental abruption (AOR = 3.3), SGA (AOR = 2.8), preterm birth (AOR = 7.1), and 5 min Apgar score < 7 (AOR = 2.4). GH significantly increased the risk of preterm birth (AOR = 1.8).

**Table 4:** Comparison of outcomes in women with gestational hypertension and pre-eclampsia using logistic regression model.

	Unaffected N (%) ^a^	GH				PE			
		N (%) ^a^	Crude OR [95% CI]	Adjusted OR ^b^ [95% CI]	*P*	N (%) ^a^	Crude OR [95% CI]	Adjusted OR ^b^ [95% CI]	*P*
Cesarean section	2038 (28.6)	99 (35.4)	1.37 [1.06, 1.75]	1.10 [0.84, 1.44]	0.48	119 (53.6)	2.88 [2.20, 3.77]	2.21 [1.66, 2.95]	<0.01
Placental abruption	75 (1.1)	2 (0.7)	0.68 [0.17, 2.77]	0.61 [0.15, 2.57]	0.50	11 (5.0)	4.91 [2.57, 9.37]	3.29 [1.57, 6.91]	<0.01
SGA	473 (6.6)	20 (7.1)	1.08 [0.68, 1.72]	1.14 [0.70, 1.85]	0.61	38 (17.1)	2.90 [2.02, 4.17]	2.81 [1.89, 4.18]	<0.01
Preterm birth	516 (7.2)	35 (12.5)	1.83 [1.27, 2.64]	1.82 [1.23, 2.68]	<0.01	87 (39.2)	8.26 [6.22, 10.98]	7.05 [5.14, 9.68]	<0.01
5 min Apgar < 7	137 (2.0)	8 (2.9)	1.48 [0.72, 3.06]	1.17 [0.55, 2.46]	0.69	16 (7.2)	3.86 [2.26, 6.60]	2.35 [1.31, 4.21]	<0.01

GH: gestational hypertension. PE: pre-eclampsia. SGA: small for gestational age (<10^th^ percentile).

^a^ Count (N) and incidence (%) of an outcome in non-exposure group (unaffected) and exposure group (PE or GH), respectively.

^b^ Adjusted for age, race, education, income, smoking, folic acid supplementation, BMI, parity, pre-eclampsia history, ART, diabetes, infant sex, and number of babies.

Supplemental analysis using pre-gravid BMI (self-reported) instead of BMI measured at recruitment showed that the effect sizes of all variables remained consistent (data not shown).

## Discussion

Among the 7633 women, the incidence rates of GH and PE were 3.7% and 2.9%, respectively. By comparing risk factors, our study identified several common risk factors for GH and PE (especially late-onset PE), including BMI ≥ 25, nulliparity, PE history, type 1 and type 2 diabetes, and twin birth. Effect sizes of PE history (RR = 14.1 for GH vs. RR = 6.4 for PE) and twin birth (RR = 4.8 for GH vs. RR = 10.3 for PE) were substantially different between GH and PE. Risk factors modify GA at delivery in women with GH and PE in similar patterns. Average GA in high-risk women is shortened compared with that in low-risk women. In subgroup analysis, early-onset PE shared some of the risk factors compared to late-onset PE, including BMI, PE history and twin birth, while folic acid supplementation showed a protective effect only for early-onset PE. With respect to outcomes, PE significantly increases the risks of adverse outcomes including cesarean section, placental abruption, SGA, preterm birth, and 5 min Apgar score < 7, while GH increases the risk of preterm birth only, with a much smaller effect size.

Our study has several limitations. First, some confounders that would potentially impact the incidence of GH or PE such as pregnancy interval [21], change in paternity [22] and renal function [23] were not available in our study. Second, in subgroup analysis, the number of cases with early-onset PE was limited. This resulted in greater random error than GH and late-onset PE subgroups. For instance, the effect of diabetes was not estimable for early-onset PE. Third, the use of BMI in early pregnancy may lead to misclassification bias although supplemental analysis using pre-gravid BMI (self-report) yielded consistent results. Fourth, ART was not specified in our study, and comparison between oocyte donation and autologous oocytes through in-vitro fertilization was not available. Fifth, the cohort was mainly comprised of white women, and the conclusions may not be generalized to other populations. Last but not least, most of the risk factors have been recognized previously, and our study might not provide valuable implications for clinical practice. Nevertheless, the study views risk factors from the perspective of statistics and epidemiology rather than clinical experience.

There are several strengths of the present study. This is among the first studies using recently introduced criteria for PE, defined by de novo hypertension and proteinuria after gestational week 20, or new onset preeclampsia-associated signs in the absence of proteinuria. Second, a new statistical approach was used to compare how risk factors modify the GA at delivery between patients with GH and PE. The method provides a new way to view the occurrence and progression of HDP from a perspective of statistics rather than physiology. Third, we excluded patients with chronic hypertension in order to ensure that GH and PE cases were diagnosed based on de novo hypertension that develops in pregnancy. Our investigators adjudicated all diagnoses by pulling medical charts, so that the quality of diagnosis was guaranteed.

Comparison of risk factors and outcomes in association with GH and PE may help to shed light on the hypothesis that GH and PE are within the same disorder spectrum with different severities. Different from previous studies [10–14], we used a novel approach to investigate and compare the risk factors [16]. The approach assumes that all women would develop HDP if the pregnancy was to continue indefinitely, and the occurrence of disease depends on competition between delivery before or after the development of HDP. GA at delivery does not reflect the time of onset of the de novo hypertension, but it indicates the zenith of severity before delivery, owing to the progressive nature of the disorders [9]. Women at high risk develop HDP at earlier stage of gestation and may need termination of pregnancy. Results of Cox regression showed that risk factors modified the average GA in women with GH and PE in similar patterns; but in each category of a risk factor, the curve for PE was left-shifted compared with curve for GH. This indicates that women who subsequently developed PE were potentially at higher risk of known or unknown factors than those who developed GH, because the former might require a shorter time to reach a zenith of the disorder before delivery. Similarly, the survival curves were also left-shifted in women at high risk compared with those at low risk.

In line with previous studies [10–14], we found that BMI (either in early pregnancy or pre-gravid) was a common risk factor for GH, early- and late-onset PE, and the effect sizes were nearly identical. A recent meta-analysis reported that overweight or obese women had approximately 2–4 fold increased PE risk compared to normal-weight women [24]. The mechanism that obesity increases GH and PE risks is not well established; but they are connected with several shared features, including oxidative stress, dyslipidemia, insulin resistance, hyperinsulinemia and impaired endothelium function. The shared pathophysiology of GH and PE has been indicated by dyslipidemia (suggesting the ongoing atherosclerotic processes) in early gestation and elevated C-reactive protein (implicating inflammation) in previous studies [25–28].

Primiparity also increases the risks of GH and PE with the same magnitude. It has been demonstrated that parous women have lower incidences of PE and eclampsia; however, this “protective” effect seems to be lost after a long (≥ 5 years) interpregnancy interval [21]. Also, change of paternity was also associated with increased risks of both GH and PE [22]. On the contrary, changing paternity reduces the risk of recurrent PE among women with PE in their first birth [29]. Although such potential confounders as interpregnancy interval and change in paternity were not available to us for analysis, findings in previous studies suggest that both GH and PE may be associated, in part, with the immunologic etiology. During pregnancy, the maternal immunological response allows maternal tolerance to the semi-allogeneic (in spontaneous pregnancies) or allogeneic (in egg-donation pregnancies) fetus; however, a defective maternofetal immune response may contribute to the development of pregnancy complications including GH and PE [30]. This “immunologic theory” is supported by epidemiologic evidences that higher incidence rates of GH and PE have been observed in ovum donor recipients compared with women conceived with autologous oocytes [31, 32].

Consistent with previous studies [11, 13], diabetes (type 1 and 2) and twin birth are also shared risk factors for GH and late-onset PE. The association between diabetes and early-onset PE was not estimable due to limited number of observations. The effect size for PE is consistent with pooled estimates in a meta-analysis (multiple birth: OR = 2.9, 2.0–4.2; insulin-dependent diabetes: OR = 3.6, 2.5–5.0) [33]. Diabetes increases the risk possibly due to microvascular changes that impair the placental perfusion, and changes in lipid metabolism [34]. Multiple birth is associated with larger placental size which leads to higher exposure to impaired placental perfusion [10]. The adverse pregnancy outcomes have a does-response relationship with the number of babies [35]. Some studies reported inconsistent results that multiple birth and diabetes are unique risk factors for PE but not for GH [10, 14]. The discrepancies between literatures may be attributed to sample size, diagnostic criteria, and statistical methodology.

We found that women with PE history were at high risks of GH and recurrent PE. The effect size for recurrent PE was similar with the pooled estimates in a meta-analysis for thirteen observational studies [33]. Surprisingly, the effect size for GH was close to early-onset PE, but was three-fold to late-onset PE. The difference between GH and late-onset PE lacks appropriate explanation. It is increasingly clear that women with history of PE, especially early-onset PE and recurrent PE, are at greater risk of developing cardiovascular disease [36–38]. It is possible that transient but severe endothelial dysfunction, observed in PE, potentiates a cascade of events that progress atherosclerosis [37]. Patterns of maternal vascular remodeling and responsiveness show a distinct vascular adaptation between early- and late-onset PE [39]. Women with early-onset PE also show higher total vascular resistance compared to women with late-onset PE postpartum [40]. Another difference in risk factors was folic acid supplementation. Folic acid showed a protective effect on early-onset PE only. This is consistent with our previous finding that the association between folic acid supplementation and PE depended on the risk profile of the women [19]. Limited sample size in early-onset PE subgroup (N = 33) might partly explain the differences, and we believe that subgroup analysis for late-onset PE generated more stable and reliable statistical results.

The effect size of twin birth for PE (both early- and late-onset) was twice to that for GH. This may be attributed to different pathophysiologic progress because the multiple gestation-related burden grows as the pregnancy continues. It is different from other risk factors that may remain unchanged during pregnancy. The inability to adequately perfuse the placenta owing to its large size or inadequate development of blood supply to the placenta is the inciting feature of PE [41]. Uteroplacental vascular resistance increases as the twin fetuses grow, and angiogenic factors released by the placenta may compromise the vascular endothelium function in a dynamic process [42]. Hence, the difference in effect size of twin birth can be explained by the underlying pathophysiologic difference between GH and PE. This hypothesis is supported by biomarker studies which show distinct cytokine profiles and placental angiogenic factors between GH and PE [28, 43]. Altered biomarkers among women with GH or PE may indicate a late manifestation of the full-blown syndrome rather than primary etiologic factors.

In summary, GH, early- and late-onset PE showed some differences with respect to risk factors, effect size, and outcomes. This indicates that the conditions may have different pathophysiology and mechanism. Survival curves showed that risk factors modified the GA in women with GH and PE in similar patterns, but the curve for PE was left-shifted compared with GH. This indicates that women who subsequently developed PE were potentially at higher risk than those who developed GH.

## Supporting information

S1 Research Data All information that identify an individual were removed from the data. (SAS7BDAT)
